# Green Ironmaking at Higher H_2_ Pressure: Reduction Kinetics and Microstructure Formation During Hydrogen-Based Direct Reduction of Hematite Pellets

**DOI:** 10.1007/s40831-024-00877-4

**Published:** 2024-07-01

**Authors:** Özge Özgün, Imants Dirba, Oliver Gutfleisch, Yan Ma, Dierk Raabe

**Affiliations:** 1 Max Planck Institute for Sustainable Materials GmbH, Max-Planck-Straße 1, 40237 Düsseldorf, Germany; 2 Institute of Materials Science, Technische Universität Darmstadt, 64287 Darmstadt, Germany

**Keywords:** Sustainability, Green ironmaking, Direct reduction, Reduction kinetics, Microstructure

## Abstract

Hydrogen-based direct reduction (HyDR) of iron ores has attracted immense attention and is considered a forerunner technology for sustainable ironmaking. It has a high potential to mitigate CO_2_ emissions in the steel industry, which accounts today for ~ 8–10% of all global CO_2_ emissions. Direct reduction produces highly porous sponge iron via natural-gas-based or gasified-coal-based reducing agents that contain hydrogen and organic molecules. Commercial technologies usually operate at elevated pressure, e.g., the MIDREX process at 2 bar and the HyL/Energiron process at 6–8 bar. However, the impact of H_2_ pressure on reduction kinetics and microstructure evolution of hematite pellets during hydrogen-based direct reduction has not been well understood. Here, we present a study about the influence of H_2_ pressure on the reduction kinetics of hematite pellets with pure H_2_ at 700 °C at various pressures, i.e., 1, 10, and 100 bar under static gas exposure, and 1.3 and 50 bar under dynamic gas exposure. The microstructure of the reduced pellets was characterized by combining X-ray diffraction and scanning electron microscopy equipped with electron backscatter diffraction. The results provide new insights into the critical role of H_2_ pressure in the hydrogen-based direct reduction process and establish a direction for future furnace design and process optimization.

**Graphical Abstract:**

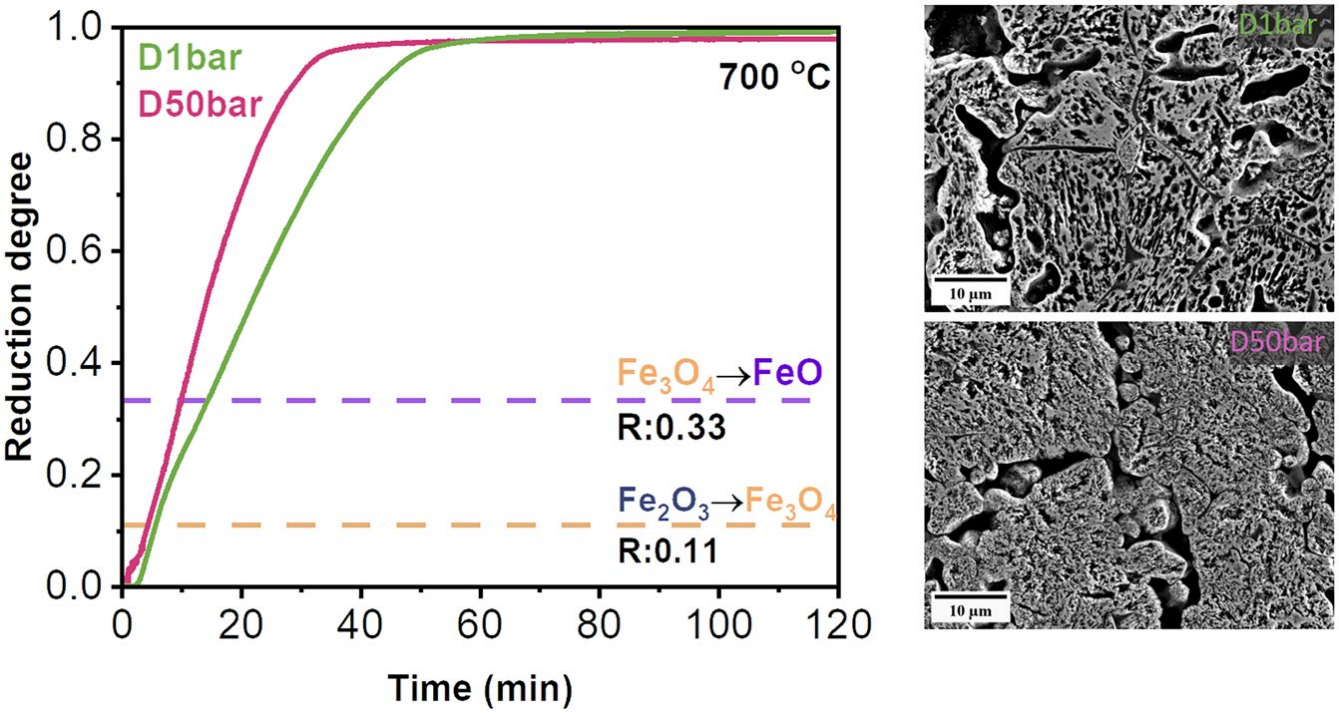

## Introduction

Steel is the foundation material of modern civilization, serving in construction, infrastructure, machinery, transportation, etc. Its massive annual production has approached 2 billion tons per year in 2023 [1]. Currently, the established integrated route of iron- and steelmaking, providing 2/3rd of the global market, proceeds in a two-step process. The first one consists of the reduction of iron oxides to pig iron, a near-eutectic Fe–C compound, in blast furnaces. The second one is steelmaking, which consists of the conversion of pig iron to steel (usually with a much lower C content of 0.01–0.4 wt%) in a basic oxygen furnace [2]. However, this integrated route is based on the use of fossil agents (i.e., coal and coke) as energy sources and reducing agents, leading to 1.9–2.2 tons of CO_2_ emissions per ton of steel. Thus, the steel industry alone accounts for ~ 8–10% of global CO_2_ emissions [2]. To reduce these immense CO_2_ emissions from the steel sector and thus cap the biggest single contributor to global warming, hydrogen is considered a promising alternative to carbon-based reductants, given that hydrogen can be produced in the required amounts by electrochemical or plasma processes using renewable electrical energy [3, 4]. In this context, hydrogen-based direct reduction (HyDR) is the most compelling technology for green ironmaking due to its high technology readiness level, that is TRL 6–8 [5]. Hydrogen-based direct reduction is a multistep solid-state reaction, where iron ores (hematite or magnetite) are gradually reduced to iron at high temperatures of 500–1100 °C [6]. H_2_ reduces the iron oxides by reacting with the chemically bound oxygen and the reaction sequence proceeds gradually from iron’s highest to its lowest oxidation state, i.e., through hematite (Fe_2_O_3_) to magnetite (Fe_3_O_4_), wüstite (Fe_1−x_O, where x indicates the deficiency of Fe in the lattice), and metallic iron (Fe). At temperatures below 570 °C, wüstite is thermodynamically unstable, and thus magnetite is directly reduced to metallic iron.

Direct reduction is a mature technology and ~ 125 million tons of steel were produced in 2021 via steam-reformed natural-gas-based and gasified-coal-based direct reduction [1]. The commercial reduction technologies used today are usually operated at elevated pressures. For example, the MIDREX and HyL/Energiron processes, using iron ore pellets (10–16 mm in diameter), are operated at 2 bar and 6–8 bar total pressure, respectively [7–10]. These operating pressures refer to the total gas pressure values of the charged reductant mixtures. In the MIDREX process hydrogen ratio in the reducing gas mixture (i.e., H_2_, CO, CH_4_, CO_2_, and H_2_O) is typically in the range of 55–80% [11]. In the MIDREX reduction method, methane (CH_4_) goes first through a gas reforming step wherein a mixture of H_2_ and CO reductants is produced in a reformer via the following reactions: CH_4_ + H_2_O → 3H_2_ + CO and CH_4_ + CO_2_ → 2H_2_ + 2CO (H_2_O and CO_2_ are obtained by collecting the off-gas of the shaft furnace). The reformed gas is then purged into the shaft furnace to reduce iron oxide [12]. In the HyL/Energiron process variants, there is no separate reformer system and the reducing gases are generated by in-situ reformation of natural gas inside the shaft furnace. The use of elevated pressure conditions facilitates the dissociation of methane over the iron ore pellets in the shaft furnace (e.g., the HyL/Energiron Zero Reformer process). The CIRCORED process is another solid-state reduction furnace variant. It is based on a fluidized bed principle and is operated at 4 bar to enable the fluidization of fine iron ore particles (50–100 µm) [7, 13–18].

In terms of the reduction kinetics, the effects of the pressure of the pure reducing gases (e.g., H_2_, and CO) [19, 20] and reducing gas mixtures (e.g., H_2_, H_2_O, CO, CO_2_, and CH_4_) [21, 22] have been investigated in the literature. It has been found that the pressure of reducing gas mixture has two major effects: (1) An increase in the absolute pressure (e.g., gas mixtures with total pressure from 1 to 3 bar containing 0.55 bar H_2_, 0.09 bar CO, 0.05 bar CO_2_ and N_2_ in balance) does not have a substantial effect on reduction kinetics [19, 22]; (2) Increasing the partial pressure of the reducing gas (e.g., ${{P_{{H_{2} \left( {or CO} \right)}} } \mathord{\left/ {\vphantom {{P_{{H_{2} \left( {or CO} \right)}} } {\left( {P_{{H_{2} \left( {or CO} \right)}} + P_{{H_{2} O\left( {or CO_{2} } \right)}} } \right)}}} \right. \kern-0pt} {\left( {P_{{H_{2} \left( {or CO} \right)}} + P_{{H_{2} O\left( {or CO_{2} } \right)}} } \right)}}$) enhances the reduction kinetics by facilitating faster counter-current diffusion of the gaseous reactants and products associated with the underlying redox reactions (e.g., increasing ${P}_{{H}_{2}}$ from 0.55 to 1.65 bar doubled the reduction rate at the initial and medium stages of reduction) [19, 23, 24]. For hydrogen-based direct reduction, it has been suggested that an increase in H_2_ pressure increased the net diffusion rate of the H_2_ through the product layer and enhanced the mass transport of the reducing gas to the reaction zone, thus improving overall reduction kinetics by up to 25% for an H_2_ pressure range of 5–35 bar [25]. The H_2_ gas pressure plays an important role not only in the reduction kinetics but also in the microstructure evolution of iron ore during reduction [3, 7, 26–28]. Specifically, the reduction behavior of iron ore pellets shifts from the classical topochemical features (at ~ 1 bar) to spatially more homogeneous reaction features, revealed by the homogeneous distribution of the partially reduced iron oxides (magnetite and wüstite) at higher pressures of H_2_ and CO gas mixtures (~ 3 bar) [21].

However, the impact of H_2_ pressure on reduction kinetics and microstructure evolution during iron ore reduction with H_2_ has not been systematically investigated and understood in terms of the underlying mechanisms. Particularly, the local reaction behavior at the microscopic scale has remained unclear so far [29–31]. These facts make it challenging to conduct pellet, gas, and process optimization for higher efficiency and faster metallization [32–36]. In the current investigation, we studied the detailed mechanisms and microstructure effects observed upon the change in H_2_ pressure, to turn efforts towards further process design from an empirical to a knowledge-based approach. We investigated the influence of pressure on the reduction kinetics of hematite pellets with pure H_2_ at 700 °C at various pressures, i.e., 1, 10, 50, and 100 bar, under static and dynamic reductant gas exposure conditions. The microstructure of partially and fully reduced pellets was characterized by combining X-ray diffraction (XRD) and scanning electron microscopy (SEM) equipped with electron backscatter diffraction (EBSD). The local porosity evolution and grain morphology of metallic iron were thoroughly characterized to better understand the effects of H_2_ pressure on the local reaction behavior. The results obtained provide new insights into the critical role of H_2_ (partial) pressure in the hydrogen-based direct reduction process and establish a direction on future pellet, furnace, and process design.

## Experimental Method

### Reduction of Hematite Pellets with Hydrogen

Commercial direct-reduction hematite pellets of 2.8 ± 0.2 g were used in this study, consisting of 0.36 wt% FeO, 1.06 wt% SiO_2_, 0.40 wt% Al_2_O_3_, 0.73 wt% CaO, 0.57 wt% MgO, 0.19 wt% TiO_2_, 0.23 wt% V, 0.10 wt% Mn, with traces of P, S, Na, and K, and Fe_2_O_3_ in balance.

The reduction of hematite pellets was performed at various pressures in two setups, namely, (1) a static gas reactor and (2) a dynamic gas reactor. In the static gas setup, hematite pellets were reduced at 700 °C for 5, 30, and 120 min in a custom-made high-pressure vessel inside a tube furnace with a chamber volume of 0.095 L [37]. The pure H_2_ gas (99.999% purity, Air Liquide) was pressurized at room temperature to reach 1, 10, and 100 bar at 700 °C. The pellets were heated up to 700 °C with a heating rate of 5 °C/min in hydrogen gas. It is worth mentioning that additional H_2_ was supplied against potential gas leakage to maintain a constant pressure of 100 bar. After reduction, the samples were cooled down to room temperature in the furnace. The reduction parameters are listed in Table 1. In the dynamic gas exposure experiments, pellets were reduced in a high-pressure thermogravimetric analysis (HP-TGA, DynTHERM, TA Instrument) setup. The gas pressures were set to be 1.3 and 50 bar (the maximal pressure allowed in the HP-TGA). The pellets were first heated to 700 °C in Ar at elevated pressures at a heating rate of 10 °C/min. When the temperature was stabilized, the gas was changed to the pressurized H_2_. The gas flows were 200, and 500 mLs/min for 1.3 and 50 bar, respectively. Real-time mass loss of hematite pellets was recorded by a magnetic suspension balance. The reduction degree (R) of the pellets was determined by Eq. (1), where ${M}_{0}$, ${M}_{f}$, and, ${M}_{t}$ are the initial mass, the instantaneous mass, and the theoretical mass after the complete reduction of the hematite pellet, respectively [38].

**Table 1:** The list of samples and their reduction conditions

Reduction condition	Sample designation	Temperature (°C)	Gas pressure (bar)	Reduction time (min)	Gas flow (mLs/min)
Static gas (interrupted tests)	S1bar5min	700	1	5	–
	S1bar30min		1	30	–
	S1bar120min		1	120	–
	S10bar5min		10	5	–
	S10bar30min		10	30	–
	S10bar120min		10	120	–
	S100bar5min		100	5	–
	S100bar30min		100	30	–
	S100bar120min		100	120	–
Dynamic gas (continuous tests)	D1bar	700	1.3	120	200
	D50bar		50	120	500

1 $$R=\frac{{M}_{0}-{M}_{f}}{{M}_{0}-{M}_{t}}$$

### Microstructural Characterization

The reduced pellets were sliced into disk-shaped samples from the middle of the pellets with a thickness of ~ 1 mm using a diamond wire saw. Subsequently, the samples were grinded using SiC papers from 320 to 4000 grits and followed by polishing using diamond suspension with a particle size of 3 µm and 1 µm and final polishing with colloidal silica suspension (OPS). The microstructure of the samples was then characterized using secondary electron (SE) and backscattered electron (BSE) imaging modes in a Zeiss Merlin scanning electron microscope. In addition, electron backscatter diffraction was employed to characterize the local distribution of the phases. The step size of the electron backscatter diffraction measurement was 50 nm and the electron backscatter diffraction data were analyzed using the software OIM Analysis™ V9. The porosity analysis was performed on 12 secondary electron images (×500 magnification, corresponding to 38,590 µm^2^ imaging area) using the software ImageJ. The inherited pores from the pelletizing process were identified to be > 95 µm^2^ in the unreduced hematite pellet. To reveal the fraction and size of the acquired pores in reduced pellet, pores with a size below 95 µm^2^ were further analyzed.

**Fig. 1:**
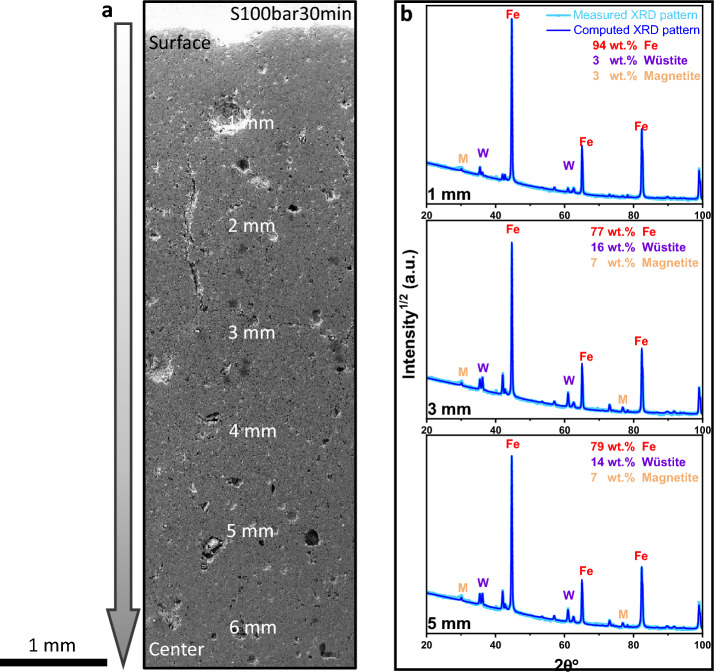
Demonstration of the phase distribution in the sample S100bar30min. **a** Secondary electron image of the sample from surface to the center. **b** X-ray diffraction profiles from a distance of 1, 3, and 5 mm below the pellet surface. All experiments were conducted at 700 °C. (M stands for magnetite, W for wüstite, and α-Fe for bcc-iron.). The values of error bars in phase analysis are smaller than 0.6 wt%

To identify the phases in the samples, X-ray diffraction analysis was employed using a Rikaku SmartLab diffractometer equipped with Cu-K_α_ radiation (λ = 1.5406 Å). The beam size was set to be 0.5 × 0.5 mm^2^. To reveal also the spatial distribution of the individual phases in the pellet samples [31], X-ray diffraction measurements were performed from the pellet surface to the center with a step size of 1 mm, as shown in Fig. 1a. The Rietveld refinement method was used to quantify the individual phases in conjunction with the Material Analysis Using Diffraction (MAUD) software, Fig. 1b [39].

## Results

### Influence of H_2_ Pressure on Reduction Kinetics Under Static Gas Condition

**Fig. 2:**
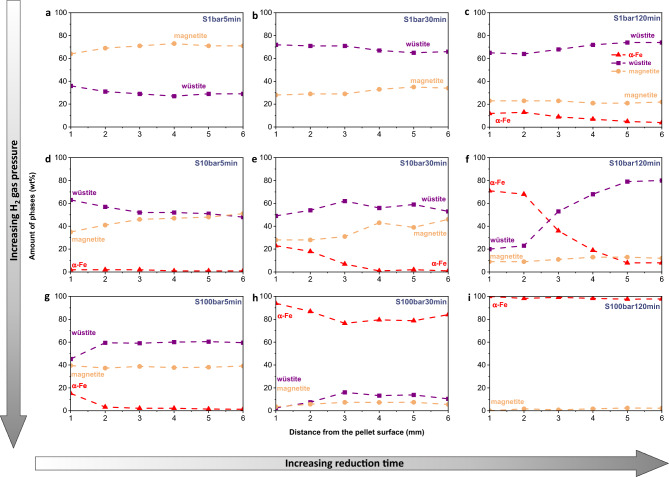
Spatial distribution of individual phases as a function of pellet diameter from the surface to the center for the pellet reduced under the static gas condition **a**–**c** at 1 bar, **d**–**f** at 10 bar, and **g**–**i** at 100 bar; **a**, **d**, **g** for 5 min, **b**, **e**, **h** for 30 min, and **c**, **f**, **i** for 120 min. The amount of phase was probed using X-ray diffraction with a beam size of 0.5 mm × 0.5 mm. All experiments were conducted at 700 °C

The spatial distribution of the magnetite, wüstite, and α-iron along the pellet diameter was probed using X-ray diffraction, as shown in Fig. 2. After 5 min of reduction, the hematite completely transformed into magnetite in all the pellets reduced at different H_2_ pressures. At the macroscopic scale, pellets exhibited the typical topochemical characteristics of reduced pellets in the solid state, as clearly revealed by the spatial gradient of individual phases along the pellet diameter. Here, the term ‘topochemical characteristics’ refers to the spatial gradient of high and low oxidation states of iron, for instance, an increasing trend in the quantity of the high oxidation state of iron from the pellet surface to the center since the reaction starts at the pellet surface and proceed through the pellet interior [31].

The comparison of the distribution of phases in the pellets reduced at different pressures for the same duration indicated that an increase in the H_2_ pressure resulted in faster reduction kinetics. For example, the pellet reduced at 1 bar for 5 min (S1bar5min) showed ~ 70 wt% magnetite and ~ 30 wt% wüstite on average in the pellet (Fig. 2a), whereas an increase in H_2_ pressure to 10 bar resulted in 48 wt% magnetite, 51 wt% wüstite, and a subtle amount of α-iron (1 wt%) in the pellet reduced for 5 min (Fig. 2d). When the H_2_ pressure was further increased to 100 bar, a higher fraction of α-iron was found in the surface region (~ 15 wt%) (Fig. 2g). Such differences strongly suggested an enhancement of the reduction kinetics when increasing the H_2_ pressure in the static gas condition. The same trend was also observed with prolonged reduction time. After 120 min, less than 10 wt% α-iron was found in the pellet reduced at 1 bar (Fig. 2c). In contrast, more than 98 wt% α-iron was observed at 100 bar (Fig. 2i), suggesting an almost completed reduction in the latter case.

### Influence of H_2_ Pressure on Microstructure Formation Under Static Gas Exposure Condition

**Fig. 3:**
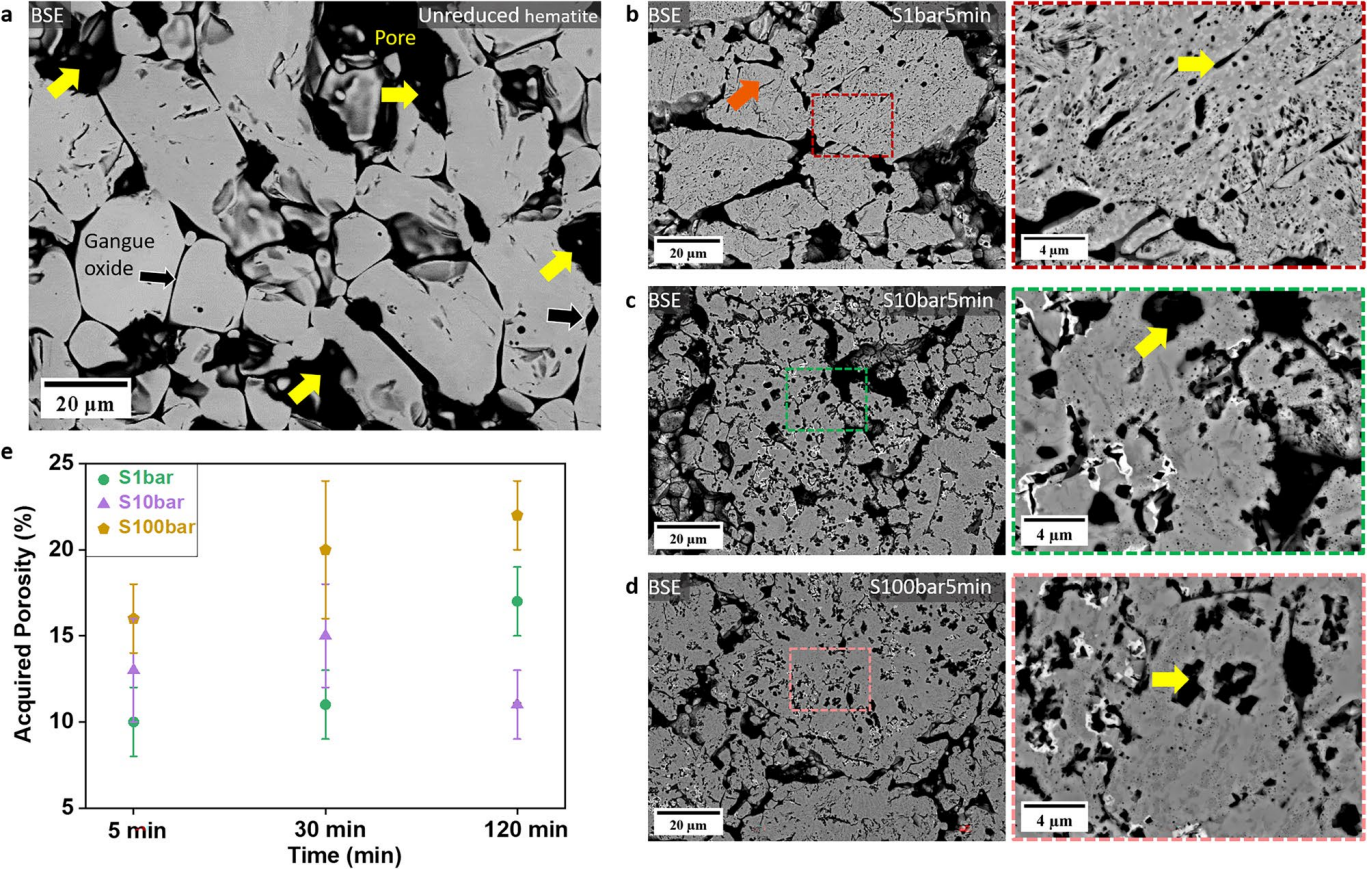
The backscattered electron image of **a** unreduced pellet and pellets reduced for 5 min at **b** 1 bar, **c** 10 bar, **d** 100 bar, and **e** evolution of acquired porosity as a function of reduction time at various H_2_ pressures (Analysis based on secondary electron images taken from the region about 2 mm below the pellet surface). All experiments were conducted at 700 °C

Figure 3a–d reveal the evolution of the pore morphology of pellets reduced at 700 °C for 5 min at 1, 10, and 100 bar H_2_ pressures, respectively. The regions marked by dark orange and yellow arrows are iron oxides (here a mixture of magnetite and wüstite) and pores, respectively, Fig. 3b. All pellets had a large amount of inherited (~ 30%) pores distributed among dense hematite grains, see Fig. 3a, as well as the acquired pores. The former were generated during palletization (high-temperature sintering of the ore fines), while the latter formed and evolved during reduction, due to the gradual removal of oxygen and the formation of cracks [26, 40]. The formation of the pores and their connectivity are critical for the overall reduction kinetics as they provide fresh oxide surfaces for chemical reactions and pathways for the outbound gaseous diffusion of the redox product gases H_2_O and CO_2_, respectively [41]. For the pellet reduced at 1 bar for 5 min (S1bar5min), elongated pores were observed within the iron oxide grains (here a mixture of magnetite and wüstite), as shown in Fig. 3b. In contrast, the sample reduced at elevated pressure values of 10 bar (Fig. 3c) and 100 bar (Fig. 3d), for 5 min revealed circular pores instead of the elongated morphology.

The evolution of the acquired porosity of pellets reduced under static gas conditions is shown in Fig. 3e. Pellets reduced at 1 bar and 100 bar exhibited a similar trend. The acquired porosity of these two pellets increased progressively to 17% (1 bar) and 22% (100 bar) with further removal of oxygen over time. Pellets reduced at 100 bar possessed a higher porosity since the reduction degree was higher (e.g., 98% for 120 min) than that of pellets reduced at 1 bar (73% for 120 min). The evolution trend of the acquired porosity in pellets reduced at 10 bar differed from those reduced at 1 and 100 bar. It was observed that the porosity was 13% in the pellet reduced at 10 bar for 5 min, and it slightly increased to 15% after hydrogen-based direct reduction for 30 min, followed by a decrease to 11% in the pellet reduced for 120 min. Such a decrease might be due to the coalescence of the acquired pores and growth into pores with a size larger than 95 µm^2^ to minimize the total interfacial energy [42], i.e. this would be a statistical effect in data analysis.

**Fig. 4:**
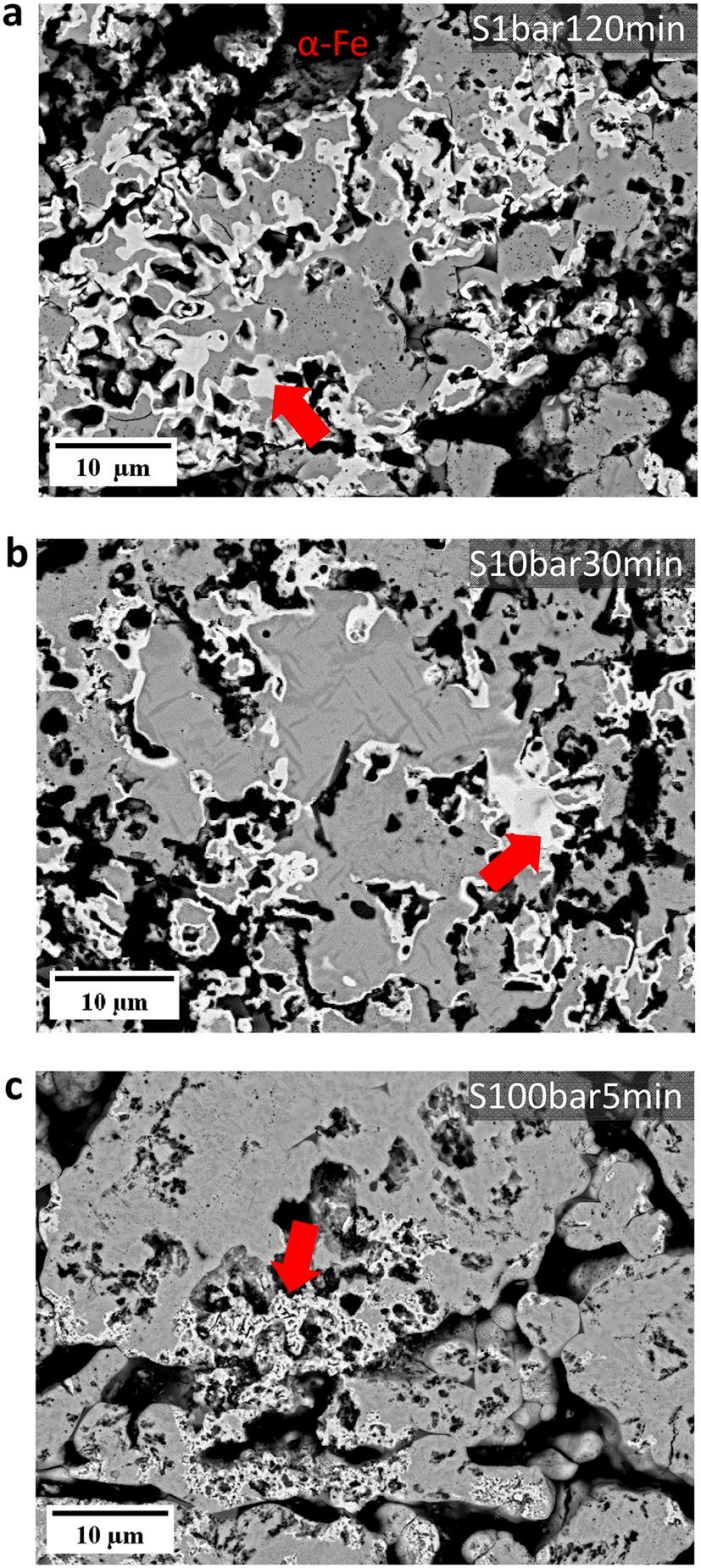
The morphology changes of metallic iron as a function of various H_2_ pressures in **a** S1bar120min, **b** S10bar30min, and **c** S100bar5min samples. All experiments were conducted at 700 °C

In addition to the change in the morphology of the pores (Fig. 3b–d), the morphology of the α-iron also evolved differently at elevated pressure. Figure 4 demonstrates the α-iron formed in the S1bar120min (Fig. 4a), S10bar30min (Fig. 4b), and S100bar5min (Fig. 4c) samples. These particular materials were selected due to their comparable α-iron fractions close to the pellet surface (~ 15% at S1bar120min, ~ 20% at S10bar30min, and ~ 18% at S100bar5min). The formation of α-iron was primarily found in the proximity of pores. In the sample reduced at 1 bar for 120 min, dense iron layers formed and encapsulated the unreduced iron oxides (Fig. 4a). A similar morphology and distribution behavior of α-iron was also observed in the sample reduced at 10 bar (Fig. 4b). In contrast, α-iron revealed a porous structure at 100 bar of H_2_ pressure (Fig. 4c).

**Fig. 5:**
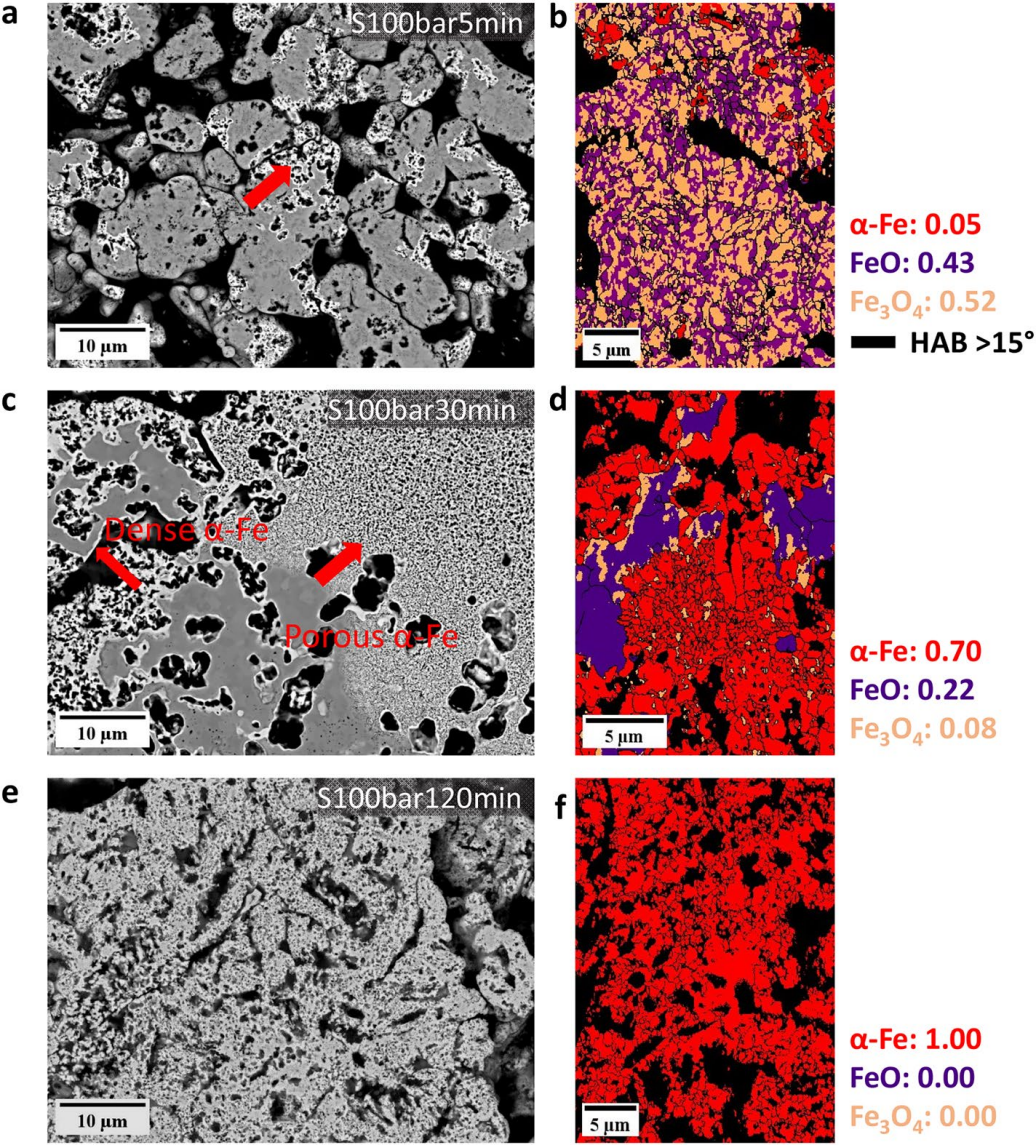
The backscattered electron image and phase map probed by electron backscatter diffraction of **a**, **b** S100bar5min, **c**, **d** S100bar30min, **e**, **f** S100bar120min pellet. All experiments were done at 700 °C. HAB stands for high-angle grain boundary

The morphology of α-iron under 100 bar H_2_ pressure evolved with reduction time, Fig. 5. After hydrogen-based direct reduction for 5 min, porous iron with 1.0 ± 0.1 µm grain size formed on the iron oxide surface. The S100bar5min sample revealed a homogeneous distribution of magnetite (52 area %) and wüstite (43 area %), deviating from the topochemical pattern (Fig. 5b). As reduction proceeded for 30 min, the iron oxide (22 area % wüstite, 8 area % magnetite) was encapsulated by ultrafine iron grains (0.5 ± 0.2 µm), Fig. 5c. It is worth noting the presence of magnetite layers between wüstite and α-iron (Fig. 5d) in the S100bar30min pellet. This phenomenon might result from the phase decomposition of wüstite into iron and magnetite during cooling since wüstite is not thermodynamically stable below 570 °C. As the hydrogen-based direct reduction proceeded, the ultrafine iron grains coarsened to 2.3 ± 0.2 µm, as depicted in Fig. 5e, f.

In summary, the H_2_ partial pressure affected the microstructure of hematite pellets in two aspects under static gas exposure conditions: (1) the acquired pores as a result of the reduction showed elongated morphology at low H_2_ pressure (1 bar), while circular pores were observed at elevated H_2_ pressure (10 bar and 100 bar); (2) Porous iron formed at high pressure (100 bar), while dense iron layers were observed at low and moderate H_2_ pressure (1 bar and 10 bar).

### Influence of H_2_ Pressure on Reduction Kinetics Under Dynamic Gas Exposure Condition

**Fig. 6:**
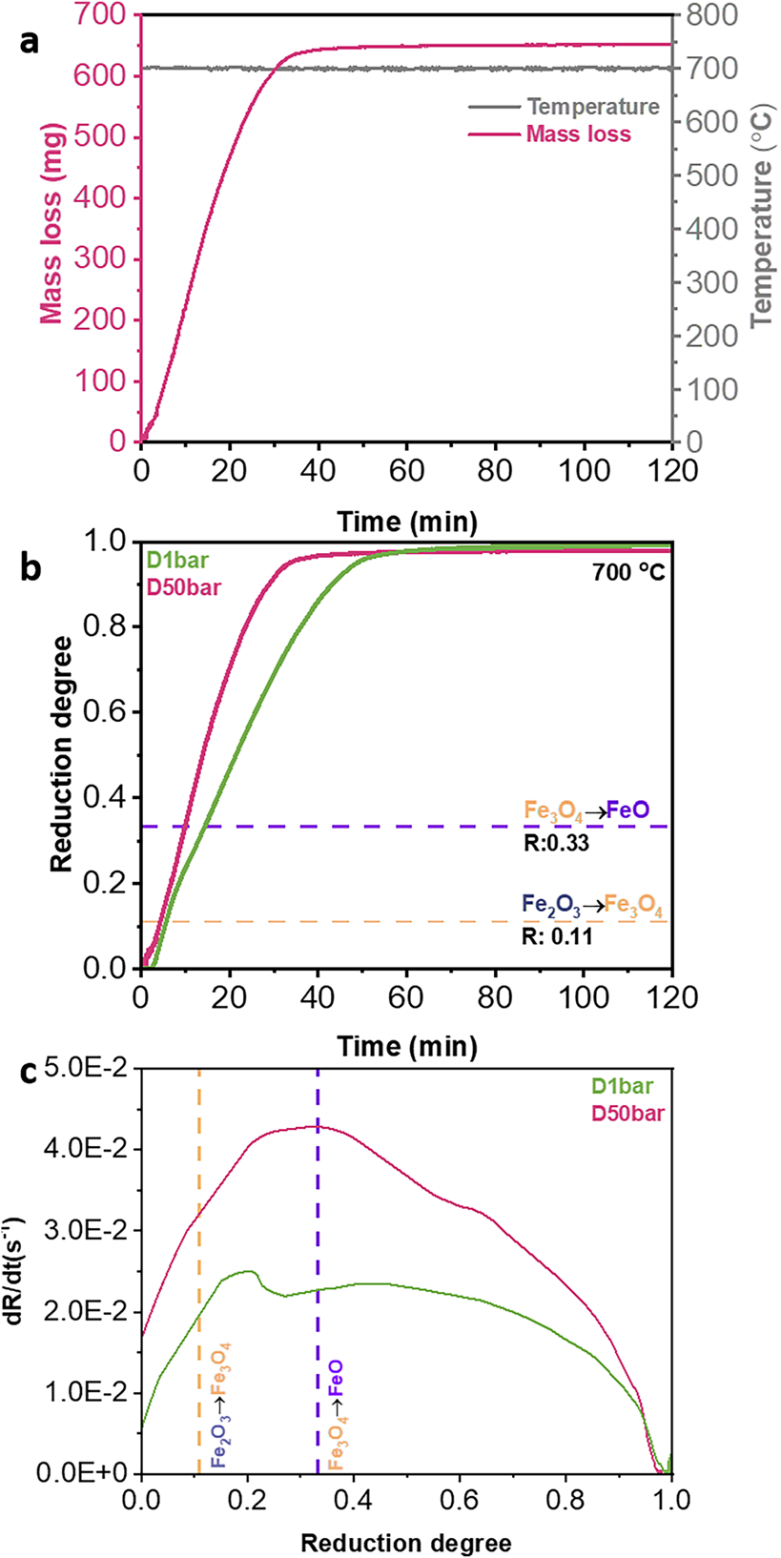
The reduction kinetics of hematite pellets reduced at 700 °C in pure H_2_ at different pressures under the dynamic gas condition. **a** Instantaneous mass loss of the pellet reduced at 50 bar, measured by thermogravimetry analysis. **b** Reduction degree (R) of the pellets reduced at different H_2_ pressure values. Yellow and purple dash lines mark the theoretically expected reduction degree from hematite to magnetite (R = 0.11) and from magnetite to wüstite (R = 0.33), respectively. **c** Plots of reduction rate (dR/dt) versus reduction degree (R). All data acquired from reduction experiments at 700 °C (Color figure online)

Figure 6 presents the reduction kinetics of pellets reduced at 1.3 and 50 bar H_2_ gas pressure under dynamic gas exposure conditions. As an example, the instantaneous mass loss of the pellet reduced at 50 bar (D50bar) is demonstrated in Fig. 6a. After purging H_2_ for approximately 1.5 min, the onset of mass loss was observed. The mass loss reached a steady state at ~ 650 mg after ~ 50 min at 700 °C, suggesting completion of reduction. Figure 6b represents the reduction degree of pellets as a function of time. At the initial stage of reduction, pellets exhibited an incubation period for 3 and 1 min at H_2_ pressure of 1.3 and 50 bar, respectively. The apparent incubation period may stem from the time needed for gas exchange within the reaction chamber, i.e., from Ar to H_2_. The reduction degree of D1bar and D50bar pellets reached ~ 0.95 after 49 and 33 min, respectively. In addition, the plots of reduction rate (dR/dt, Fig. 6c) revealed a higher reduction rate in the pellet reduced at 50 bar compared with that reduced at 1 bar. In both cases, the reduction rate decreased gradually in the stage when wüstite started to transform into α-iron. Such a decrease was supposed to be due to the limited removal kinetics of oxygen through the dense iron layers, encapsulating the remaining wüstite [26, 31].

### Microstructural Evolution Under the Dynamic Gas Condition

**Fig. 7:**
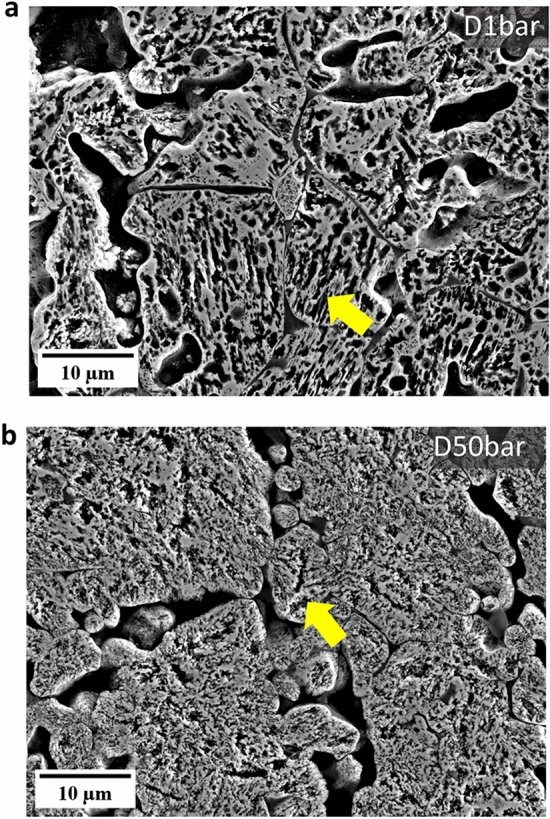
Secondary electron images of the sample reduced at **a** 1.3 bar, and **b** 50 bar under the dynamic gas condition. Yellow arrows indicate pores. Experiments were done at 700 °C (Color figure online)

Figure 7 shows the microstructure of the reduced pellets at 1.3 and 50 bar H_2_ gas pressure at 700 °C for 120 min under the dynamic gas condition. The pore morphology in the reduced pellets changed from an elongated shape at ambient pressure (Fig. 7a) to a more circular one at 50 bar (Fig. 7b), similar to the morphology observed under static gas exposure. This finding was further quantitatively supported by a decrease in the aspect ratios of acquired pores from 1.83 (at 1.3 bar) to 1.64 (at 50 bar) with an increase in H_2_ pressure. The pellets reduced at 1.3 bar and 50 bar showed similar porosity values, i.e., 21.0 ± 4.0% at 1.3 bar and 19.0 ± 5.0% at 50 bar. In addition, the grain size of the reduced iron was significantly finer, i.e., dropping in average size from 9.0 ± 1.0 µm (at 1.3 bar) to 1.0 ± 0.8 µm (at 50 bar) when treated under an increased H_2_ gas pressure.

## Discussion

### Influence of H_2_ Pressure on Reduction Kinetics

Table 2 shows the average fractions of the individual phases in the spherical pellets. The spatial distribution curves for magnetite, wüstite, and α-iron along the pellet radius (Fig. 2) were fitted individually using polynomial functions available in the OriginPro 2022 software. Subsequently, the polynomial equations were integrated for the spherical volume to calculate the phase fractions in the spherical pellets.

**Table 2:** The quantity of α-iron, wüstite, and magnetite in the pellets reduced under the static gas reduction conditions at 700 °C

Sample	Metallic iron (wt%)	Wüstite (wt%)	Magnetite (wt%)
S1bar5min	–	29	71
S1bar30min	–	67	33
S1bar120min	6	71	23
S10bar5min	1	51	48
S10bar30min	7	64	29
S10bar120min	18	70	12
S100bar5min	1	38	61
S100bar30min	80	12	8
S100bar120min	99	–	1

During hydrogen-based direct reduction, the reaction occurs in several steps: (1) Transport of H_2_ molecules to the reaction front (e.g., pellet and surface of open pores); (2) Dissociation of H_2_ into H atoms on the iron oxide and metallic iron surfaces [43, 44]; (3) Adsorption of H atoms; (4) Oxygen removal via H reaction with the oxygen in the iron oxides, i.e., formation of H_2_O, iron cations, and anion vacancies; (5) Desorption of H_2_O from the reaction surface; (6) Diffusion of H_2_O and transport with the gas stream.

Next, the influence of H_2_ pressure on these individual steps will be discussed for reduction kinetics under static gas exposure conditions. During the early stage of hydrogen-based direct reduction, the kinetics is controlled by a mixture of mass transfer of gas molecules to the reaction front and the chemical reaction [24]. At high pressure, the collision frequency of H_2_ molecules with the iron oxide surface increases [45]. In this study, thus, the reduction kinetics at the initial stage was supposed to be most pronounced at 100 bar. Regarding steps (2) and (3), Li et al. [46] investigated the dissociation and adsorption behavior of H_2_ on hematite and iron at various pressure levels (1 to 1000 bar) and temperatures (from room temperature up to 627 °C) by applying a combination of density functional theory calculations and statistical thermodynamics. They suggested that high pressure facilitates the dissociation and adsorption of H_2_ molecules. The increase in the quantity of absorbed H_2_ molecules on wüstite and hematite with increasing H_2_ pressure is also supported by the work of Cheng et al. [47], who arrived at a similar conclusion by using ReaxFF molecular dynamics simulations. Step (4) can be assessed according to Le Chatelier’s principle [48], considering that the overall reaction of hydrogen-based direct reduction is Fe_2_O_3(s)_ + 3H_2(g)_ → 2Fe_(s)_ + 3H_2_O_(g)_. Le Chatelier’s principle suggests that variations in total pressure should not significantly impact the rate equilibrium of a reaction when the number of moles of the gas molecules in both reactants and products is the same. This in turn means that the rate of the chemical reaction should not be affected by the absolute pressure. Steps (5) and (6) can be assessed considering the counter-current diffusion of H_2_ (inward) and H_2_O (outward) molecules through the reaction front (pore or pellet surface). Assuming that the same amount of hematite was reduced into iron at different H_2_ pressures, the amount of the produced H_2_O should be the same. Since the amount of H_2_ molecules in a confined volume is higher at 10 and 100 bar (i.e., ×10 for 10 bar, and ×100 for 100 bar) than that at 1 bar, the partial pressure of H_2_ (${{P_{{H_{2} }} } \mathord{\left/ {\vphantom {{P_{{H_{2} }} } {\left( {P_{{H_{2} }} + P_{{H_{2} O}} } \right)}}} \right. \kern-0pt} {\left( {P_{{H_{2} }} + P_{{H_{2} O}} } \right)}}$) will be highest at 100 bar, followed by 10 bar and finally 1 bar. This condition results in a faster outward flux of H_2_O at 100 bar than at 10 and 1 bar due to the higher concentration gradient of H_2_ and H_2_O between the reaction surface and the gas stream.

Also, the change in partial pressure of H_2_ has a substantial effect on the thermodynamics of the reaction. The Gibbs energy of a solid–gas reaction can be expressed along Eq. (2);

2 $$\Delta G=\Delta {G}^{0}+RT\text{ln}Q$$

wherein $\Delta {G}^{0}$ is the standard Gibbs free energy of the reaction and $Q$ is the instantaneous reaction quotient. For the reaction Fe_2_O_3(s)_ + 3H_2(g)_ → 2Fe_(s)_ + 3H_2_O_(g)_, $Q=\frac{{{(p}_{{H}_{2}O})}^{3}}{{{(p}_{{H}_{2}})}^{3}}$, ranging from zero (i.e., for pure H_2_) to infinity (i.e., for pure H_2_O). ${p}_{{H}_{2}}$ and ${p}_{{H}_{2}O}$ are the partial pressure of H_2_ and H_2_O, respectively. An increase in the value of ${p}_{{H}_{2}}$ thus lowers $Q$. Hence, the overall thermodynamic driving force of the chemical reaction increases (i.e., decreasing $\Delta G$ to a more negative value). In addition, the kinetics of the removal of oxygen increases with increasing H_2_ partial pressure [49], as expressed by Eq. (3):

3 $$ R_{0} = \phi^{f} P_{{H_{2} }} \left[ {1 - exp\left( {{{\Delta G} \mathord{\left/ {\vphantom {{\Delta G} {RT}}} \right. \kern-0pt} {RT}}} \right)} \right], $$

where ${R}_{o}$ is the rate of oxygen removal from the iron oxide surface and ${\phi }^{f}$ is the apparent chemical reaction constant for the forward reaction (Fe_2_O_3(s)_ + 3H_2(g)_ → 2Fe_(s)_ + 3H_2_O_(g)_). Since the value of ${p}_{{H}_{2}}$ in the reducing gas is highest at 100 bar, followed by 10 and 1 bar, the rate of oxygen removal follows the same sequence for the rate of reaction at 100, 10, and 1 bar. Consequently, the enhanced reaction rate at elevated pressures of H_2_ gas under static gas conditions is attributed to the increase in the partial pressure of H_2_.

Under dynamic gas conditions, the pellet reduced at 50 bar exhibited a higher reduction rate compared with the pellet reduced at 1.3 bar. All the aforementioned aspects, relating pressure to reaction rates, also apply to the dynamic reduction experiments. Similar to the static gas exposure conditions, the partial pressure of H_2_ in the reaction chamber is higher at 50 bar compared with 1.3 bar, resulting in an enhancement of the overall reduction kinetics. An additional contribution to the enhanced reduction kinetics at 50 bar H_2_ gas pressure may stem from the higher H_2_ flow rate at 50 bar (500 mLs/min) than at 1.3 bar (200 mLs/min) [50]. Compared with the reduction at 1 and 10 bar under static conditions, the faster reduction kinetics of the reduction conducted at 1.3 bar under dynamic reductant exposure is attributed to the continuous hydrogen supply to the reaction chamber.

### Influence of H_2_ Pressure on the Pellets’ Microstructure Formation

In this study, our findings highlighted two major effects of pressure on the microstructure formation and its temporal evolution during hydrogen-based direct reduction. First, the morphology of the acquired pores changed from an elongated to a more circular shape, as pressure increased from ambient to elevated pressures, as depicted in Figs. 3b–d and 7a, b. Second, the morphology of the iron altered from dense iron layers found at H_2_ pressures of 1 and 10 bar to porous iron at 100 bar (Fig. 4). Several studies have investigated the correlation between the morphology of iron and the composition of the reducing gas [51–55]. In these studies, it has also been observed that the morphology of metallic iron depends on the partial pressure of the reducing gas (i.e., H_2_ or CO). Increasing the partial pressure of H_2_ results in the formation of porous iron, while an increase in the partial pressure of H_2_O causes denser iron growth, which is associated with the effect of the partial pressure of H_2_ on the rate of oxygen removal.

During the reduction of the wüstite to iron, the local concentration of iron increases on the wüstite surface as a result of the removal of oxygen. The increase in local concentration of iron in the surface regions causes a chemical potential gradient between the outer surface and the bulk wüstite inside of the pellet that drives the diffusion of iron towards the bulk wüstite (Fe_1−x_O; 0.83 < 1 − x < 0.95). As reduction proceeds, the concentration of iron ions in the wüstite increases, and eventually wüstite becomes saturated with iron ions (Fe_1−x_O; (1 − x) approaches 0.95). The accumulation of excess iron results in iron clusters and iron nucleation events. Iron nuclei grow with the incoming flux of reduced iron at the iron-wüstite interfaces [54].

**Fig. 8:**
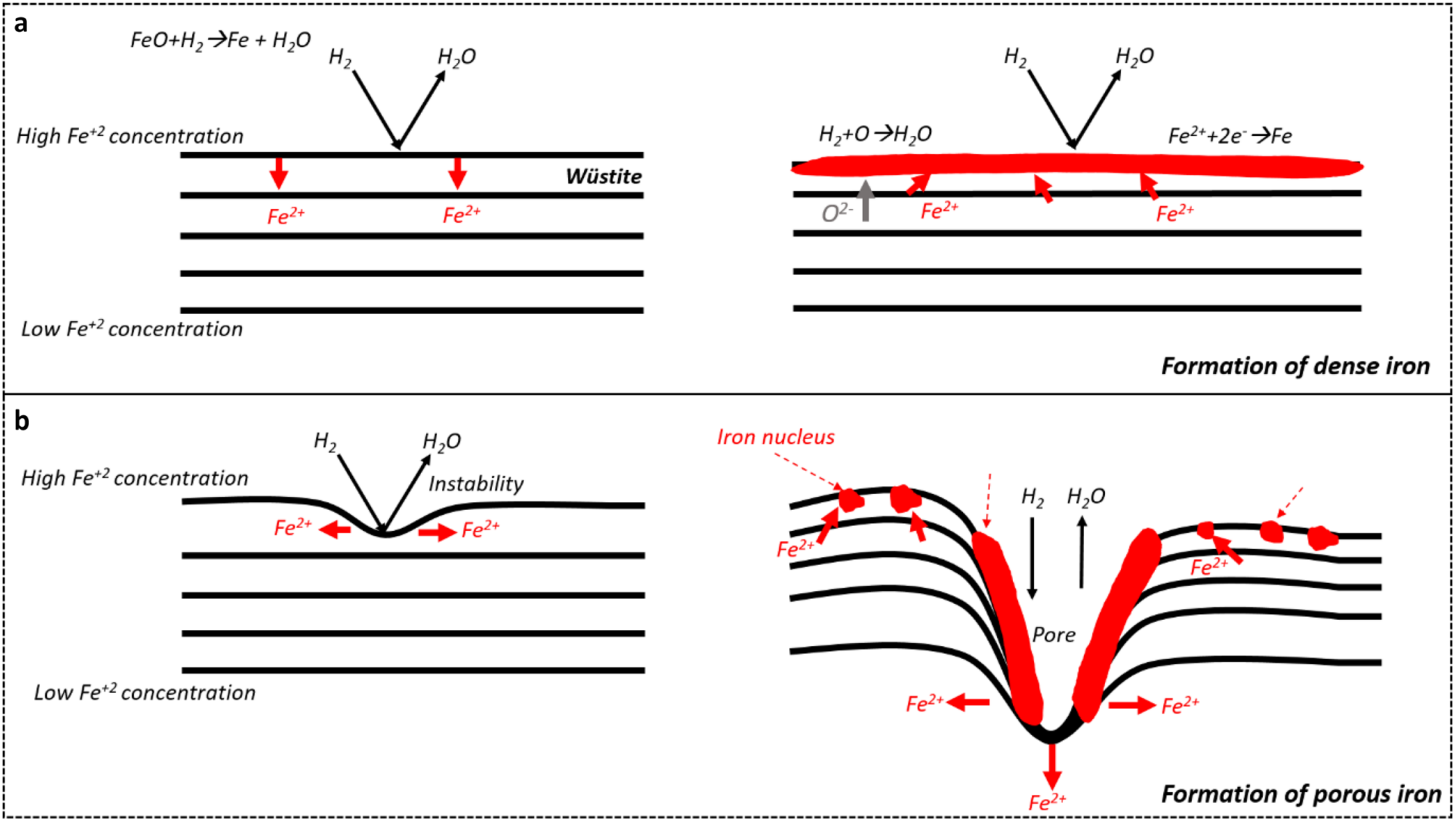
Schematic illustration of the formation of **a** dense and **b** porous α-iron depending on the respective rates of pressure-dependent oxygen removal and iron diffusion [58]

The morphology of α-iron (i.e., dense layer or porous iron) is controlled by the competition between the rate of oxygen removal from the surface and the rate of iron diffusion from the wüstite towards the metallic iron phase [56, 57]. Figure 8 schematically shows the formation of the dense iron layer and the porous iron during hydrogen-based direct reduction. When the diffusion rate of iron is higher than the rate of oxygen removal, excess iron readily diffuses in a direction perpendicular to the wüstite surface. The concentration of excess iron exhibits a homogeneous distribution throughout the wüstite surface. In this case, a planar wüstite surface is maintained. When the iron ions are saturated inside the wüstite, excess iron nucleates homogeneously on the surface and a dense iron layer forms (Fig. 8a). When the removal rate of oxygen is faster than the diffusion rate of iron from wüstite to the metallic iron phase, the morphology of the iron alters. The presence of a perturbation (i.e., distortion on the surface at the atomic/molecular level) induces an instability in the form of an alternating sequence of concave and convex features that can protrude into the wüstite (Fig. 8b). This instability results in the rejection of iron in the direction parallel to the wüstite surface. Consequently, the concentration of iron on the tip of the perturbation becomes less than that of the planar surface. Due to the lower concentration of iron, the rate of chemical reaction on the tip of such a perturbation feature becomes faster than that of a planar surface. This process continues until the planar surface becomes unstable, resulting in the formation of porous iron [58] (Fig. 8b). The removal rate of oxygen is determined by the combination of temperature and reducing gas composition, while the diffusion rate of iron is determined only by temperature and by the local chemical potential gradient. As indicated in Eq. (3), the removal rate of oxygen at 100 bar is faster compared with 1 and 10 bar, yet the diffusion rate of the iron remains the same. Therefore, the formation of the porous iron at 100 bar H_2_ pressure can be attributed to the faster removal of oxygen from the iron oxide compared with the unchanged diffusion rate of the iron, while the formation of the dense iron layer at 1 and 10 bar is attributed to the slower removal rate of oxygen compared with iron diffusion.

Furthermore, changes in the H_2_ pressure causes also a change in pore morphology, from elongated pores at ambient pressure to pores with circular shapes at elevated pressures. The literature suggests two mechanisms that could initiate the formation of porous iron on the wüstite surface: the breakdown (or bursting) of a dense iron layer and the formation of an instability on the oxide surface [54]. The former results from the water production and accumulation at the iron/wüstite interface when hydrogen diffuses through the dense iron layer and reacts with oxygen at the interface. If a void exists at the interface, H_2_O or H_2_ gas bubbles form and gradually expand the void. Once the gas pressure at the interface becomes larger than the pressure of the reducing gas, the breakdown of the dense iron layer occurs and forms a pore [59]. The latter case is the same as the formation of porous iron due to the faster removal rate of oxygen from the iron surface [58]. The formation of elongated pores at D1bar and S1bar5min samples may stem from the instability formation and its growth during the transition from wüstite to the α-iron phase. At the H_2_ pressure of 1 bar, the rate of the reduction at the initial stage (for 5 min) can be much faster due to the high initial hydrogen partial pressure and the removal rate of oxygen can suppress the diffusion rate of iron [57].

The grain size of α-iron in fully reduced pellets, namely S100bar120min and D50bar samples, is 2.3 ± 0.2 and 1.0 ± 0.8 µm, respectively. Moreover, the grain size of α-iron decreased from 1.0 ± 0.1 µm (S100bar5min) to 0.5 ± 0.1 µm (S100bar30min) at 100 bar H_2_ pressure under the static gas conditions. Such ultrafine grain size of α-iron indicates a high density of iron nucleation events on the wüstite surface during hydrogen-based direct reduction when exposed to high H_2_ pressure conditions. In this case, α-iron is likely to grow by transporting iron from the saturated wüstite adjacent to these nuclei. This scenario suggests that the morphology of the pores is dominated by the nucleation of iron. Consequently, small radii and a large number of pores form around the fine iron grains.

## Conclusion

In this study, we studied the hydrogen-based direct reduction of commercial polycrystalline hematite pellets at elevated H_2_ pressure under both static (1, 10, and 100 bar) and dynamic (1.3 and 50 bar) gas exposure conditions at 700 °C, to understand the effects of H_2_ pressure on the reduction kinetics and microstructure formation. The main conclusions are summarized as follows:

1. The hematite pellets exhibited increasing reduction kinetics with an increase in H_2_ pressure under both static gas exposure conditions (1, 10, and 100 bar) and dynamic gas exposure conditions (1.3 and 50 bar).
2. Under both static and dynamic reduction conditions, the morphology of the pores in the reduced pellets changed from an elongated structure at ambient pressure to a circular structure at an elevated H_2_ pressure. The elongated pores form due to an instability formation and its growth during the reduction of wüstite to α-iron when the diffusion rate of iron is slower than the removal rate of oxygen. At elevated pressure, a high number density of iron nucleation results in the formation of a large number of pores with small radii.
3. The reduction of iron ore at a hydrogen gas pressure of 100 bar represents an extreme scenario regarding the current direct reduction furnace operations at the industrial scale. Nonetheless, our observations have unveiled reduction kinetics and microstructure formation (i.e., ultra-fine grains) in the direct reduced iron under such extreme conditions. This study could also inspire applications related to the hydrogen-based redox reactions of iron oxides at high pressure, such as for catalytical applications, as well as the fabrication of ultrafine microstructure via hydrogen-based direct reduction.
4. Pellets reduced at an H_2_ pressure of 1 and 10 bar, respectively, exhibited dense iron formation on wüstite as a consequence of the low partial pressure of H_2_ and the slow reduction kinetics. The slower oxygen removal compared with the diffusion rate of iron resulted in the formation of a dense iron layer. In contrast, elevated H_2_ pressure resulted in fast oxygen removal compared to the unchanged iron diffusion from the reaction interface towards the iron nuclei favors the formation of porous iron structures.
5. The H_2_ pressure plays an important role in the reduction kinetics and microstructure formation during hydrogen-based direct reduction of iron oxides. An increasing H_2_ pressure increases the partial pressure of H_2_, which promotes faster reduction kinetics. This fact should be considered for the design of industrial reactors.
